# Modulating Neuronal Activity Produces Specific and Long-Lasting Changes in Numerical Competence

**DOI:** 10.1016/j.cub.2010.10.007

**Published:** 2010-11-23

**Authors:** Roi Cohen Kadosh, Sonja Soskic, Teresa Iuculano, Ryota Kanai, Vincent Walsh

**Affiliations:** 1 Department of Experimental Psychology and Oxford Centre for Functional MRI of the Brain, University of Oxford, Oxford OX1 3UD, UK; 2 University College London Medical School, Department of Life Sciences, University College London, London WC1E 6BT, UK; 3 Institute of Cognitive Neuroscience, University College London, London WC1N 3AR, UK

## Abstract

Around 20% of the population exhibits moderate to severe numerical disabilities [1–3], and a further percentage loses its numerical competence during the lifespan as a result of stroke or degenerative diseases [4]. In this work, we investigated the feasibility of using noninvasive stimulation to the parietal lobe during numerical learning to selectively improve numerical abilities. We used transcranial direct current stimulation (TDCS), a method that can selectively inhibit or excitate neuronal populations by modulating GABAergic (anodal stimulation) and glutamatergic (cathodal stimulation) activity [5, 6]. We trained subjects for 6 days with artificial numerical symbols, during which we applied concurrent TDCS to the parietal lobes. The polarity of the brain stimulation specifically enhanced or impaired the acquisition of automatic number processing and the mapping of number into space, both important indices of numerical proficiency [7–9]. The improvement was still present 6 months after the training. Control tasks revealed that the effect of brain stimulation was specific to the representation of artificial numerical symbols. The specificity and longevity of TDCS on numerical abilities establishes TDCS as a realistic tool for intervention in cases of atypical numerical development or loss of numerical abilities because of stroke or degenerative illnesses.

## Highlights

► Brain stimulation to the parietal cortex can enhance or impair numerical abilities ► The effects were specific to the polarity of the current ► The improvement in numerical abilities lasts up to 6 months ► The brain stimulation affected specifically the material that was recently learned

## Results and Discussion

Dalton, Keynes, Gauss, Newton, Einstein, and Turing are only a few examples of people who have advanced the quality of human life and knowledge through their exceptional numerical abilities. At the other end of the scale, up to 6.5% of the population struggles with even basic numerical understanding, a disability termed Developmental Dyscalculia [3]. An even higher percentage (15% to 20% of the population) has less-specific numerical difficulties, which nevertheless impose significant practical, educational, and, consequently, employment obstacles [2, 10], and a further percentage loses their numerical competence during the life span as a result of stroke or degenerative problems [4]. The negative impact of numerical difficulties on everyday life is manifested in the lack of progress in education, increased unemployment, reduced salary and job opportunities, and additional costs in mental and physical health [2, 11, 12].

At the neuronal level, studies have shown that difficulties or expertise with numbers are associated with functional and anatomical anomalies of the right parietal lobe, as compared to the normal population [13–18]. In addition, the right parietal lobe has been suggested to be important for the development of intact numerical understanding during infancy and early childhood [19, 20]. At the behavioral level, cognitive and developmental studies have shown that automatic numerical processing and mapping of numbers into space are important indices of the number sense abilities [7, 21].

Automatic numerical processing can be assessed with a numerical Stroop paradigm [22, 23]. In this paradigm, subjects are presented with two numerical stimuli on the computer screen and are required to compare the stimuli according to their physical size. The stimuli can be incongruent (e.g., a physically large 2 and a physically small 4), neutral (e.g., a physically small 2 and a physically large 2), or congruent (e.g., a physically small 2 and a physically large 4). Congruity effects reflect automatic numerical processing: longer reaction times for incongruent trials in comparison to congruent trials. Such an effect with symbolic numbers characterizes competent numerical ability [22, 23], whereas a negligible effect, if any, is a cognitive signature of adults with numerical difficulties [7, 24] or healthy children at the beginning of the first grade [22, 23].

A number-to-space paradigm probes the close relation between visuospatial processes and numerical representation [25]. In this task, subjects are asked to map a number on a physical line [26]. Mastering numerical information is characterized by a linear mapping of numbers onto a physical line [8, 9]. In contrast, young children, as well as indigenous tribes who have little or no formal mathematical education, map the numbers in a logarithmic fashion [8, 9]. Another effect that numerate adults show is a systematic spatial bias toward the larger number, whereas children show a bias toward the small number [27]. In adults, this effect is likely to be due to a spatial bias toward the larger magnitude as a result of an overestimation of the lateral extent closer to the larger digit. In children, the opposite effect is likely to be due to ordinal influence that preceded cardinality at earlier developmental stages.

We combined transcranial direct current stimulation (TDCS), a noninvasive brain stimulation technique [6, 28], with a learning paradigm of artificial digits [29], analogous to the methodology frequent in language studies [30], to investigate the causal link between the parietal lobes and the development of numerical automaticity and number-space interaction. During TDCS, a weak current is applied constantly over time to enhance (anodal stimulation) or reduce (cathodal stimulation) the excitation of neuronal populations, with maximal effect on the stimulated area beneath the electrodes [6, 28]. Animal studies have shown that the long-lasting effects are protein synthesis dependent and accompanied by modifications of intracellular cyclic AMP and calcium levels, and they therefore share some features with long-term potentiation (LTP) and long-term depression (LTD) [6, 28]. Magnetic resonance spectroscopy in humans found that the molecular changes involved reduction in spontaneous neural activity of GABAergic (anodal stimulation) and glutamatergic (cathodal stimulation) activity after motor cortex stimulation [5].

**Figure 1:**
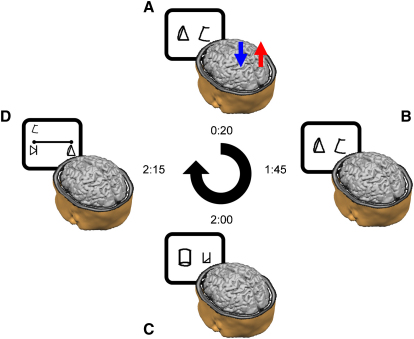
A Schematic Outline of the Experimental Design in a Typical Daily Session (A) TDCS was delivered for 20 min from the start of the training. In this case, anodal stimulation was applied to the right parietal lobe (red arrow), whereas cathodal stimulation was delivered to the left parietal lobe (blue arrow). (B) The training continued after the termination of the stimulation. (C and D) Once the training ended, the subjects performed the numerical Stroop task (C) and the number-to-space task (D). The time next to each image reflects the elapsed time from the beginning of the daily session until its termination in a cumulative fashion.

Over 6 days, 15 healthy adults learned the association between nine arbitrary symbols without knowing the quantity that had been assigned to them (see Figure S1 available online). At the beginning of each training day, when the learning phase started, a weak current (1 mA) was applied to the subjects' left and right parietal lobes for 20 min. Following the learning phase, which lasted for around 90–120 min, we assessed the subjects' newly created number sense with the numerical Stroop task and the number-to-space task with learned digits (Figure 1; Figures S2 and S3). We examined the performance as a function of three conditions: (1) the right anodal-left cathodal (RA-LC) group received anodal stimulation to the right parietal lobe and cathodal stimulation to the left parietal lobe; (2) the right cathodal-left anodal (RC-LA) group received cathodal and anodal stimulation to the right parietal lobe and the left parietal lobe, respectively; (3) the sham group received stimulation to the left and right parietal lobes that ceased after 30 s. The sham stimulation produces a sensation that is indistinguishable from the nonsham stimulation condition but that has no excitatory effect on the neuronal populations [6, 28, 31, 32].

We found that during numerical learning, anodal stimulation to the right parietal lobe and cathodal stimulation to the left parietal lobe (RA-LC group) caused better and more consistent performance in both numerical tasks. In contrast, the opposite configuration, anodal stimulation to the left parietal lobe and cathodal stimulation to the right parietal lobe (RC-LA group), led to underperformance, comparable to that observed in young children or indigenous tribes with rudimentary numerical skills (i.e., RA-LC group > RC-LA group = children [8, 9, 22, 27]). Sham stimulation led to a performance that fell between both stimulation groups.

**Figure 2:**
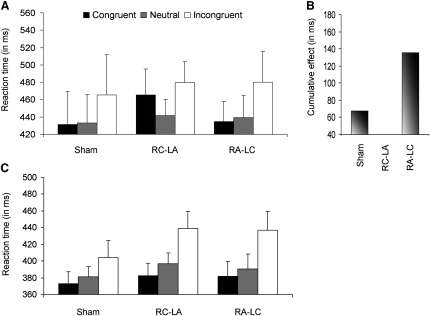
The Congruity Effect for the Artificial Digits, the Cumulative Congruity Effect over Training, and the Congruity Effect for Everyday Digits for the Sham, RC-LA, and RA-LC Groups in the Numerical Stroop Task The data of the artificial digits for each group are averaged across the sessions that showed a significant congruity effect (three sessions for the RA-LC group, two sessions for the sham group, and five sessions for the RC-LA group; note that the latter group showed an abnormal congruity effect that was not changed as a function of learning), and the raw data, which includes RTs in each session for each group, are presented in Table S1. (A) Whereas the RA-LC group and the sham group showed a typical congruity effect, the RC-LA group showed an abnormal effect that mirrored the performance of children at the age of 6 years and might reflect perceptual rather than semantic interference [22]. (B) The cumulative congruity effect demonstrates the emergence of a consistent automatic numerical processing already from the fourth day for the RA-LC group (p = 0.005, Table S1), whereas it occurred only later for the sham group (p = 0.049, Table S1) and did not appear for the RC-LA group. (C) All groups showed a consistent and typical congruity effect for everyday digits (p = 0.00009; group x congruity interaction, p = 0.46), as reflected by slower RTs for the incongruent condition versus the congruent condition. Data are mean ± standard error (SE) of the mean. Note the different scaling in each panel. For a description of the task, see Figure S2.

During the numerical Stroop task, the development of automaticity over time differed among the groups, as indicated by a significant three-way interaction between group, session, and congruity (F(16,96) = 1.85, p = 0.035, Table S1). Further analysis revealed that the RA-LC group showed an interaction between congruity and training. This interaction was due to a consistent congruity effect (43–50 ms) that was already present from the fourth training day (F(2,8) = 10.81, p = 0.005), indicating automatic numerical processing. In contrast, the RC-LA group showed an abnormal effect (F(2,8) = 5.67, p = 0.03). A quadratic trend analysis (incongruent > neutral < congruent) explained 87% of the variance (F(1,4) = 11.36, p = 0.03), indicating that this effect was due to faster reaction times (RTs) for the neutral condition in comparison to the congruent and incongruent conditions (congruent versus incongruent, p = 0.3). The sham group failed to show a significant interaction between congruity and training (F(8,32) = 1.76, p = 0.12). However, it seems that, in contrast to the RC-LA group, which did not show a typical congruity effect, and the RA-LC group, which showed a consistent congruity effect already from the fourth day (fourth day congruity effect in the sham group = 10 ms, p = 0.6), a typical congruity effect emerged for the sham group on the fifth and sixth training days (F(2,8) = 4.52, p = 0.049) (Figure 2A and Table S1).

**Figure 3:**
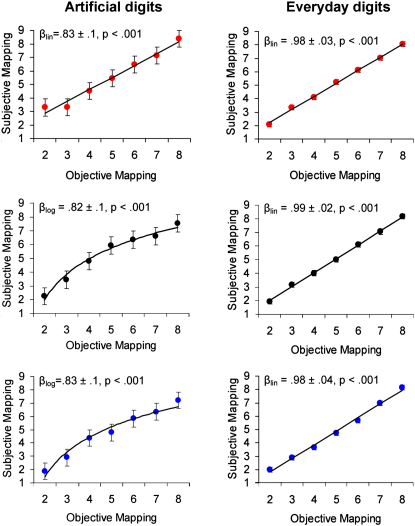
Average Location of Artificial Digits on the Horizontal Segment, Shown Separately for Artificial Digits in the Left Column, Everyday Digits in the Right Column, and Type of Stimulation β represents the selection of the best weight, whether it was logarithmic (β_log_) or linear (β_lin_), in stepwise regression analysis with linear and logarithmic predictors. Data are mean ± SE of the mean. The first row reflects the performance of the RA-LC group (red circles), the middle row reflects the performance of the sham group (black circles), and the bottom row presents the performance of the RC-LA group (blue circles). Whereas the performance with artificial digits was affected by the type of brain stimulation and showed a linear fit only for the RA-LC group, the performance with everyday digits was independent of the type of brain stimulation and showed a linear fit for all the groups. For a description of the task, see Figure S3.

Brain stimulation also affected the performance in the number-to-space task. We examined whether the mapping of the number into space follows a linear or logarithmic scale. Previous studies suggested that a log-to-linear shift might occur as a result of exposure to critical educational material or culture-specific devices such as rulers or graphs [9]. However, all studies that have documented the log-to-linear shift involved populations that showed linear mapping due to extensively learned material (i.e., the digits 1–9 that are familiar from schooling) and/or symbolic knowledge of quantity [8, 9]. The current paradigm allowed us to reveal that brain stimulation can induce a performance that is characterized by linear fit independent of exposure to critical educational material or culture-specific devices. Namely, at the end of the learning phase, a logarithmic function was the best predictor in the regression analysis for the sham group and the RC-LA group, whereas linear function characterized best the RA-LC group (Figure 3).

In addition, as indicated by a main effect for group, a rightward shift toward the large number was observed for the RA-LC group (mean = 0.59) and to a lesser degree for the sham group (mean = 0.25), a finding that characterizes adults' performance with everyday digits. In contrast, a leftward shift, which is associated with children's performance [27], was observed for the RC-LA group (mean = −0.27; F(2,12) = 5.2, p = 0.023; linear trend analysis [RA-LC > sham > RC-LA] explained 98% of the variance).

To examine whether TDCS affected more general perceptual or cognitive abilities, we asked the subjects on the last day of testing to perform the same tasks with everyday digits (Figures S2 and S3). The performance in these tasks with everyday digits was not modulated by the type of brain stimulation (all p > 0.2). Specifically, the subjects showed a normal congruity effect (F(2,24) = 14.1, p = 0.00009), which did not vary between the groups (p = 0.46, Figure 2B), and the linear scale showed the best fit to their performance, independent of group (Figure 3).

Six months after the end of the training, we contacted the participants from the RA-LC group to examine whether their adult-like performance on the tasks with artificial digits persisted. All but one of the participants was available. In the numerical Stroop task, the participants showed a significant congruity effect, as indicated by slower RTs for the incongruent versus neutral and congruent (p = 0.04). This performance was very similar to the performance on the last day of training 6 months earlier (interaction between congruity and time, p = 0.53; congruity effect of 44 ms at the end of training versus 36 ms after 6 months). In the number-to-space task, the participants showed a positive correlation between their current mapping and their performance 6 months before (r = 0.83, p = 0.02), and their performance was still best characterized by a linear function (β = 0.71 ± 0.13, p < 0.001).

Previous studies have used transcranial magnetic stimulation to the parietal lobe during numerical tasks to solely impair numerical abilities (for reviews, see [33, 34]). Although this knowledge is important for our understanding of brain organization and the brain-behavior relationship, transient impairment of an ability does not have the same, major implications as improving an ability (e.g., rehabilitation, cognitive enhancement). In contrast, the current results show that noninvasive brain stimulation can not only impair such capacities but can also enhance numerical abilities with remarkable longevity. Namely, during numerical learning, we selectively enhanced or impaired the development of automatic numerical processing and the interaction between number and space, which are critical indices of numerical abilities [7, 8].

The observed polarity effect is likely to stem from stimulating the right parietal lobe, which has been previously shown to correlate with the level of math abilities [13–18] and to be crucial for intact automatic numerical processing [24]. We can be confident that the parietal lobes are the focus of our stimulation effects because of the increased current density under the site of the electrodes [6, 28]. Nevertheless, future studies are needed that will investigate the effects of DC (Supplemental Discussion).

TDCS has been shown to affect the cellular and molecular mechanisms that are involved in LTP and/or LTD [5, 28, 31, 35]. Previous studies have pinpointed the effect of DCS to several minutes after stimulation onset [31], and in order to achieve more selective effects, it is therefore important to modulate neuronal activity via cognitive tasks prior to the brain stimulation onset [36]. Therefore, it is not surprising that the current results were highly specific to the learned material rather than to general functions such as visuospatial abilities, attention, or working memory (for further discussion, see Supplemental Discussion). In addition, TDCS did not affect the learning process itself, which might be subserved by nonparietal areas [37, 38], or the automaticity of number processing and the mapping of number into space with everyday digits. This dissociation between artificial digits and everyday digits supports the view that numbers can be represented by multiple representations [39], which has further implications for theories in numerical cognition, education, and rehabilitation.

Our findings are important because they establish TDCS as a tool for intervention in cases of atypical numerical development or loss of numerical abilities due to stroke or degenerative illnesses. To date, no pharmacological interventions have been found that could target numerical cognition directly without holding substantial side effects for other domains, such as attention [40]. Therefore, the specificity of the current findings makes the use of TDCS attractive in the field of rehabilitation of developmental and acquired disorders in numerical cognition.

## Experimental Procedures

### Participants

Fifteen right-handed university students (20–22 years old) were randomly assigned to the RA-LC group, RC-LA group, or sham group.

### Procedure

The study consisted of six sessions for each subject. The sessions lasted ∼120 min each (including electrode placement, a learning phase, and a testing phase) and were distributed over a 7 day period. Each subject attended one session per day apart from a break after the fourth day. The experiments for all the subjects started between 9 am and 6 pm.

### Tasks

The first session consisted only of the learning task, because this session also included additional participant briefing regarding the experiment, the stimulation method, and health screening. Subjects were instructed to refer to meaningless symbols (i.e., the artificial digits) as representing various magnitudes. In each trial, two symbols appeared on the computer screen, one symbol in the left visual field, and the other in the right visual field. In each trial, subjects chose the side of the display with the symbol they thought had a larger magnitude by pressing the P or Q keys on the keyboard. They were asked to respond as quickly as possible but to avoid mistakes. After each trial, a visual feedback was provided. Each learning session included 1584 trials, which were divided into 11 blocks. The learning task was the first task to be done in all six sessions. The performance of each participant was assessed by fitting the performance using a power law function (Supplemental Experimental Procedures).

Sessions 2–6 included both a numerical Stroop task (Figure S2) and the number-to-space task (Figure S3). In the numerical Stroop task, pairs of artificial digits appeared on the screen in the same manner as in the learning task, but the symbols were different in physical size. Subjects were instructed to choose the physically larger symbol by pressing either the P or Q button as quickly and accurately as possible.

In the number-to-space task, subjects mapped symbols onto a horizontal line displayed on the computer screen. The symbols corresponding to numbers 1 and 9 were placed at the left and the right of the line, respectively (Figure S3). Subjects were instructed to place the remaining symbols on this line according to their magnitude.

On the last day, after the completion of the above-mentioned tasks, the same numerical Stroop task and the number-to-space task, with the exception of everyday digits as stimuli, were additionally included (for further details about the task and design, see Supplemental Experimental Procedures).

### TDCS

Direct current was generated by a Neurocomm stimulator (Rogue Resolutions) and delivered via a pair of identical, square scalp electrodes (3 × 3 cm) covered with conductive rubber and saline-soaked synthetic sponges (Supplemental Experimental Procedures).

## Supplemental Information

Document S1. Supplemental Experimental Procedures, Supplemental Discussion, One Table, and Three Figures
