# Supplementary material for: Modulating Neuronal Activity Produces Specific and Long-Lasting Changes in Numerical Competence
Source: Curr Biol. 2010 Nov 23;20(22):2016–20. doi: 10.1016/j.cub.2010.10.007 (PMC2990865; doi:10.1016/j.cub.2010.10.007)
Supplement: Document S1. Supplemental Experimental Procedures, Supplemental Discussion, One Table, and Three Figures [file mmc1.pdf]

Current Biology, Volume *20*

Supplemental Information

Modulating Neuronal Activity Produces Specific and Long Lasting Changes in Numerical Competence

Roi Cohen Kadosh, Sonja Soskic, Teresa Iuculano, Ryota Kanai, and Vincent Walsh

Supplemental Experimental Procedures

Subjects were informed that the experiment was designed to investigate effects of TDCS on cognition but were kept blind as to the specific relevance to numerical cognition and to the type of stimulation they were receiving.

None of the participants reported significant neurological or psychiatric disorders. The study was approved by the local ethics committee and informed written consent was obtained for every subject before the start of each session.

Stimuli

Nine artificial digits were arbitrarily assigned to the numbers 1-9 (Figure S1) [1]. Subjects were unaware that these symbols represented numbers as was confirmed by a debriefing at the end of the experiment.

Learning Task

Each trial began with a fixation point (in white ink) for 300 ms at the centre of a black computer screen. Three hundred ms after the fixation disappeared two symbols (vertical visual angle of 2.63°) appeared on the computer screen, one symbol in the left visual field, and another in the right visual field. The center-to-center distance between the two digits subtended a horizontal visual angle of 9.7°. The pair appeared and remained in view until the participant pressed a key (but not for more than 5 sec). Visual feedback ("Correct Answer"/"Mistake"/"No Response") was provided for every trial for 500 ms. A new trial began 200 ms after the feedback. Each learning session was divided into 11 blocks of trials, each block consisting of 144 symbol pair comparisons (trials) that included 18 comparisons for each adjacent pair. The presentation in each block appeared in a random order. A training block with 48 trials was performed at the beginning of the task. Participants were instructed to choose the symbol they thought had a larger magnitude in each pair. They were asked to respond as quickly as possible but to avoid mistakes and to indicate their choices by pressing one of two keys (i.e., P or Q on the keyboard) corresponding to the side of the display with the selected member of the digit pair. The correct answer appeared an equal number of times on the right and left sides and all pairs appeared

equally often. Participants were provided with the average reaction time of the correct answers and percentage of errors after one third, two thirds and at end of each block. The learning task was the first task to be done in all six sessions.

The performance of each participant was assessed by fitting the performance using the following power law function (see Supplemental Data) [2]:

$$RT = B^*(N)^{-C}$$

where RT represents the mean reaction time in a given block, B is the performance in time on the first block (N = 1), N the number of the block and C is the slope of the line (i.e., the learning rate).

Numerical Stroop Task

The vertical visual angles of the symbols were 2.2° or 2.75°. Subjects were instructed to choose the *physically* larger symbol by pressing either P or Q buttons as quickly and accurately as possible. While all the possible adjacent pairs were used (e.g., 1-2, 2-3, 3-4) in the learning phase, here, only not-adjacent pairs were used (e.g., 1-3, 2-4, etc.) and congruent, incongruent, and neutral conditions were included in order to examine the generation of mental numerical representation [1]. In a congruent pair the numerically larger digit was also physically larger. In a neutral pair the digits differed only in the relevant dimension. In an incongruent pair the numerically larger digit was physically smaller. As the artificial digits that were the equivalent to the numbers 1 and 9 received the same classification during the learning phase (small, and large, respectively), they were not included in the analysis [1]. The three conditions appeared the same number of times, with the right answer appearing equal times on the right and left sides and all pairs appearing equally often. No feedback was given on the performance in this task.

Number-to-Space Task

Symbols to be mapped appeared above the right and left end of the line in a randomised order to avoid any bias in responses that might arise due to stimulus location [3]. Each symbol appeared 3 times at each location, making 42 line bisection trials in total for each session. No feedback was given on the performance in this task.

Analysis

For the analyses of the learning task, the numerical Stroop tasks, and the number-to-space task (only for the calculation of bias), the dependent variables were subjected to an analysis of variance (ANOVA) with all the relevant factors (e.g., congruity, session, learning rate) as within-subject factors, and group as a between-subject factor. For the analysis of the linear and logarithmic functions we used stepwise regression.

TDCS

For all groups at the beginning of the stimulation the current was increased slowly during the first 15 sec to the stimulation threshold (1mA) (ramp-up), and at the end of the stimulation the current was decreased slowly to 0mA during last 15 sec (ramp-down). Between the ramp-up and

ramp-down constant direct current (1mA) was delivered for 20 minutes at the beginning of each session (i.e. at the beginning of the learning task). The latter type of stimulation was not applied to the sham group in line with a previous described method [4]. Specifically, previous studies showed that after 30 sec of stimulation (15 sec of ramp-up followed by 15 sec of ramp-down) both TDCS and sham produce sensations of comparable quality, minimal discomfort and duration. Moreover, neither group is able to distinguish between TDCS and sham sessions [4].

The current was delivered through a pair of saline soaked sponge electrodes. Electrodes were positioned over the left and right parietal lobes according to the 10-20 EEG procedure on the sites corresponding to P3 and P4 respectively. We chose to place the cathodal electrode on the parietal lobe, and not on the prefrontal cortex, so as not to affect the mechanisms that might relate to learning, and feedback/reward which was provided during the learning phase [5, 6]. Other brain areas might be also involved in visual, semantic or numerical processing (occipital lobes, temporal lobes) and therefore were excluded [7]. In addition, the placement of the electrodes over both parietal lobes increases the specificity of the type of stimulation to each lobe, and increased its effect by increasing the current density [8].

Although stimulation ended during the learning task, electrodes were kept in place until task completion in order to avoid participant bias. The same set up applied for all groups and the subjects were unaware of not receiving full stimulation. All subjects reported a slight tingling sensation at the onset of the stimulation, which diminished rapidly due to habituation. No other discomforts or adverse effects were reported.

Supplemental Discussion

Specific vs. Non-Specific TDCS Effect

Our contention was to examine the effect of TDCS on the artificial digit processing. We therefore decided to display the equivalent in everyday digits only at the end of the experiment to avoid pointing the participants towards strategies that might lead to 'contamination' of this representation [7]. However, one might argue that the lack of similar effects with everyday digits was due to the longer time that had elapsed since the termination of the TDCS. We find this scenario unlikely. First, the effect of RA-LC was still present after 6 months. This excludes the possibility that the TDCS effect was limited to ~90 min and therefore did not affect the task with the everyday digits. Second, the effect of TDCS should have covered the time in which the everyday digits were presented. Previous studies showed that repeated TDCS with similar intensity, session duration and number duration have a long-lasting effect (1 week [9]; 1 month [10]; 2 weeks, [11]; 3 weeks [12]; 3 months [13]). Third, we ran another control task. This task is a reverse numerical Stroop task. Instead of processing the physical size and ignoring the numerical value as is commonly done in this task [14-20], we asked of participants to process the numerical value and ignore the physical size [21]. In this case the physical size is processed automatically, and a congruity effect is observed independent of the numerical competence (e.g., [22-24]). As with the other control tasks, this task took place at the end of the last day. Importantly, as it involved artificial digits, it took place before the other control task. All subjects did this task right after the learning phase, before the numerical Stroop task with the artificial digits. If TDCS did induce some non-specific effect, then this task should have shown some

effects for group, or an interaction between group and congruity. This was not the case (all Fs<1, all ps>.6). Notably, we observed a significant congruity effect (F(2,24)=4.5, p=.022, congruent, 968 ms; neutral, 999 ms; incongruent, 1066 ms). Together these results support the specificity of the TDCS in the current study.

Electrode Placement

In our study, enhancement occurred when the anodal electrode was placed over the right parietal lobe, while impairment occurred after cathodal stimulation to the same area. This finding is in line with previous studies that observed improvement after anodal stimulation and impairment after cathode stimulation [25-27]. However, it is entirely possible that the stimulation of the contralateral area contributed to the observed effect by modulating interhemispheric interactions/inhibitions [28].

Table S1, Related to Figure 2. Reaction Times in the Numerical Stroop Task for Each Group in Each Session

			2nd session	3rd session	4th session	5th session	6th session
RC-LA	Congruent	RT	519	473	483	428	424
		SEM	52	24	40	29	20
	Neutral	RT	487	441	465	412	401
		SEM	33	29	33	15	13
	Incongruent	RT	561	464	499	437	436
		SEM	45	25	39	22	12
RA-LC	Congruent	RT	513	447	**430**	**433**	**441**
		SEM	41	27	**25**	**37**	**13**
	Neutral	RT	523	447	**443**	**435**	**440**
		SEM	44	20	**22**	**37**	**20**
	Incongruent	RT	524	448	**480**	**476**	**485**
		SEM	52	22	**34**	**47**	**35**
sham	Congruent	RT	593	461	456	**447**	**415**
		SEM	51	20	24	**51**	**26**
	Neutral	RT	510	466	438	**442**	**424**
		SEM	33	12	19	**36**	**30**
	Incongruent	RT	581	470	466	**495**	**436**
		SEM	44	17	18	**65**	**31**

Bold numbers represent days in which normal congruity effect was observed. RT=reaction time; SEM= standard error of mean.

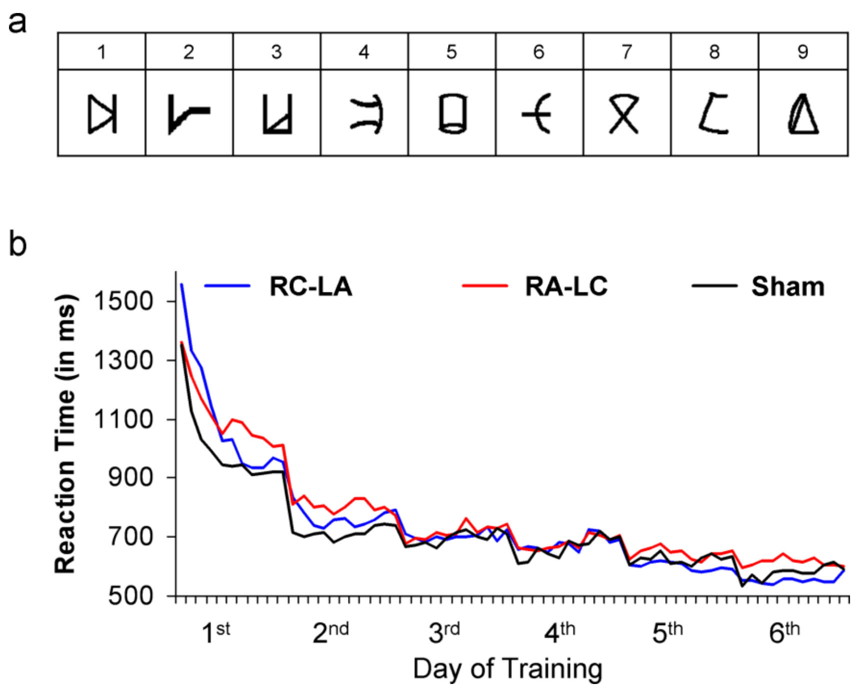

Figure S1, Related to Figure 1. Stimuli Used in the Experiment and Learning Functions for the Different Groups

A) The artificial digits used as stimuli and their equivalent in everyday digits.

B) Learning function for right cathodal, right anodal, and sham group. The improvement in the learning task over days was modelled using a power law function (see *Methods and Materials*). Non-linear regression showed an equivalent fit for all three groups (RC-LA, R=.92; RA-LC, R=.88; sham, R=.85; p=.46). In addition, the speed of learning and the reaction time for the first block were similar among the three groups (all ps>.33).

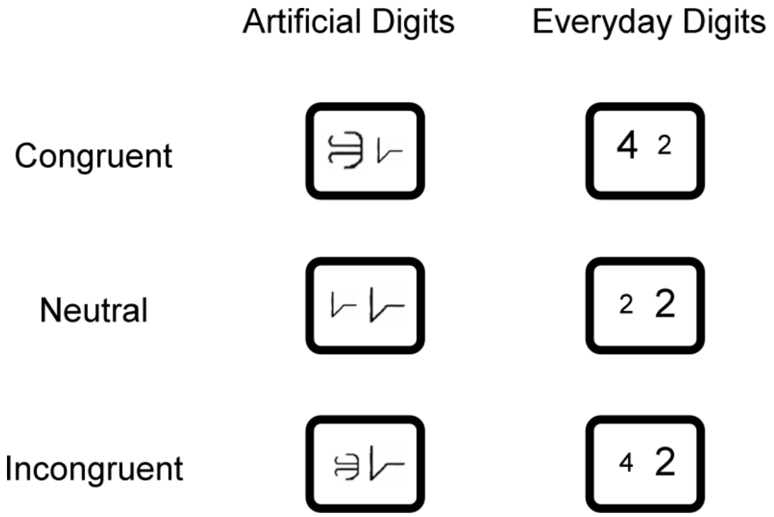

Figure S2, Related to Figure 2. An Example of the Conditions in the Numerical Stroop Task with Artificial Digits (Left Column) and Everyday Digits (Right Column)
A subject was required to determine which of the two stimuli is physically larger. The stimuli presented may be congruent, neutral or incongruent in relation to their physical size and semantic value.

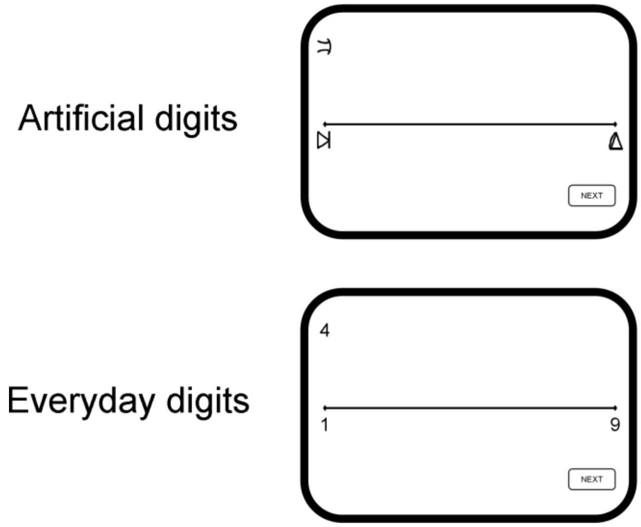

Figure S3, Related to Figure 3. Number-to-Space Task with Artificial Digits (Top) and Everyday Digits (Bottom)
Subjects were asked to map a symbol, which appeared randomly at the left upper corner, as in the current example, or at the right upper corner, on the physical line. Subjects were instructed to place each symbol on this line according to their magnitude in relation to the presented symbols at the edges of the line.

Supplemental References

1. Tzelgov, J., Yehene, V., Kotler, L., and Alon, A. (2000). Automatic comparisons of artificial digits never compared: Learning linear ordering relations. J. Exp. Psychol. Learn. Mem. Cogn. *26*, 103-120.
2. Newell, A., and Rosenbloom, P. (1981). Mechanisms of skill acqusition and the law of practice. In Cognitive skills and their acquisition, J.R. Anderson, ed. (Hillsdale, NJ: Erlbaum), pp. 1-55.
3. Nichelli, P., Rinaldi, M., and Cubelli, R. (1989). Selective spatial attention and length representation in normal subjects and in patients with unilateral spatial neglect. Brain Cogn. *9*.
4. Gandiga, P.C., Hummel, F.C., and Cohen, L.G. (2006). Transcranial DC stimulation (tDCS): a tool for double-blind sham-controlled clinical studies in brain stimulation. Clin. Neurophysiol. *117*.
5. Albert, N.B., Robertson, E.M., and Miall, R.C. (2009). The resting human brain and motor learning. Curr. Biol. *19*, 1023-1027.
6. Duncan, J. (2001). An adaptive coding model of neural function in prefrontal cortex. Nat. Rev. Neurosci. *2*, 820-829.
7. Cohen Kadosh, R., and Walsh, V. (2009). Numerical representation in the parietal lobes: Abstract or not abstract? Behav. Brain Sci. *32*, 313-373.
8. Nathan, S.S., Sinha, S.R., Gordon, B., Lesser, R.P., and Thakor, N.V. (1993). Determination of current density distributions generated by electrical stimulation of the human cerebral cortex. Electroencephalogr. Clin. Neurophysiol. *86*, 183-192.
9. Baker, J.M., Rorden, C., and Fridriksson, J. (2010). Using transcranial direct-current stimulation to treat stroke patients with aphasia. Stroke *41*, 1229-1236.
10. Boggio, P.S., Rigonatti, S.P., Ribeiro, R.B., Myczkowski, M.L., Nitsche, M.A., Pascual-Leone, A., and Fregni, F. (2008). A randomized, double-blind clinical trial on the efficacy of cortical direct current stimulation for the treatment of major depression. Int. J. Neuropsychopharmacol. *11*, 249-254.
11. Boggio, P.S., Nunes, A., Rigonatti, S.P., Nitsche, M.A., Pascual-Leone, A., and Fregni, F. (2007). Repeated sessions of noninvasive brain DC stimulation is associated with motor function improvement in stroke patients. Restor. Neurol. Neurosci. *25*, 123-129.
12. Fregni, F., Gimenes, R., Valle, A.C., Ferreira, M.J., Rocha, R.R., Natalle, L., Bravo, R., Rigonatti, S.P., Freedman, S.D., Nitsche, M.A., Pascual-Leone, A., and Boggio, P.S. (2006). A randomized, sham-controlled, proof of principle study of transcranial direct current stimulation for the treatment of pain in fibromyalgia. Arthritis. Rheum. *54*, 3988-3998.
13. Reis, J., Schambra, H.M., Cohen, L.G., Buch, E.R., Fritsch, B., Zarahn, E., Celnik, P., and Krakauer, J., W. (2009). Noninvasive cortical stimulation enhances motor skill acquisition over multiple days through an effect on consolidation. Proc. Natl. Acad. Sci. USA *106*, 1590-1595.
14. Henik, A., and Tzelgov, J. (1982). Is three greater than five: The relation between physical and semantic size in comparison tasks. Mem. Cogn. *10*, 389-395.
15. Tzelgov, J., Meyer, J., and Henik, A. (1992). Automatic and intentional processing of numerical information. J. Exp. Psychol. Learn. Mem. Cogn. *18*, 166-179.

16. Schwarz, W., and Ischebeck, A. (2003). On the relative speed account of the number-size interference in comparative judgment of numerals. J. Exp. Psychol. Hum. Percept. Perform. *29*, 507-522.
17. Szucs, D., and Soltesz, F. (2007). Event-related potentials dissociate facilitation and interference effects in the numerical Stroop paradigm. Neuropsychologia *45*, 3190-3202.
18. Cohen Kadosh, R., Cohen Kadosh, K., Linden, D.E.J., Gevers, W., Berger, A., and Henik, A. (2007). The brain locus of interaction between number and size: A combined functional magnetic resonance imaging and event-related potential study. J. Cogn. Neurosci. *19*, 957-970.
19. Cohen Kadosh, R., Henik, A., and Rubinsten, O. (2008). Are Arabic and verbal numbers processed in different ways? J. Exp. Psychol. Learn. Mem. Cogn. *34*, 1377-1391.
20. Ganor-Stern, D., and Tzelgov, J. (2008). Across-notation automatic numerical processing. J. Exp. Psychol. Learn. Mem. Cogn. *34*, 430-437.
21. Besner, D., and Coltheart, M. (1979). Ideographic and alphabetic processing in skilled reading of English. Neuropsychologia *17*, 467-472.
22. Girelli, L., Lucangeli, D., and Butterworth, B. (2000). The development of automaticity in accessing number magnitude. J. Exp. Child Psychol. *76*, 104-122.
23. Rubinsten, O., Henik, A., Berger, A., and Shahar-Shalev, S. (2002). The development of internal representations of magnitude and their association with Arabic numerals. J. Exp. Child Psychol. *81*, 74-92.
24. Rubinsten, O., and Henik, A. (2006). Double dissociation of functions in developmental dyslexia and dyscalculia. J. Educat. Psychol. *98*, 854-867.
25. Utz, K.S., Dimova, V., Oppenlander, K., and Kerkhoff, G. (2010). Electrified minds: Transcranial direct current stimulation (tDCS) and Galvanic Vestibular Stimulation (GVS) as methods of non-invasive brain stimulation in neuropsychology—A review of current data and future implications. Neuropsychologia *48*, 2789-2810.
26. Nitsche, M.A., Cohen, L.G., Wassermann, E.M., Priori, A., Lang, N., Antal, A., Paulus, W., Hummel, F., Boggio, P.S., Fregni, F., and Pascual-Leone, A. (2008). Transcranial direct current stimulation: State of the art 2008. Brain Stimul. *1*, 206-223.
27. Miniussi, C., Cappa, S., Cohen, L., Floel, A., Fregni, F., Nitsche, M., Oliveri, M., Pascual-Leone, A., Paulus, W., Priori, A., and Walsh, V. (2008). Efficacy of repetitive transcranial magnetic stimulation/transcranial direct current stimulation in cognitive neurorehabilitation. Brain Stimul. *1*, 326-336.
28. Cappelletti, M., Barth, H., Fregni, F., Spelke, E.S., and Pascual-Leone, A. (2007). rTMS over the intraparietal sulcus disrupts numerosity processing. Exp. Brain Res. *179*, 631-642.
